# Identifying Factors Contributing to Dropouts in a Pilot Telenutrition Weight-Loss Program: A Qualitative Study

**DOI:** 10.1089/tmr.2024.0071

**Published:** 2024-12-11

**Authors:** Noura M. S. Eid, Noor A. Hakim, Najlaa M. Jawad, Sarah N. Alsharif, Soaad Alsulami, Khulud A. Almalki, Dana S. Aljohani

**Affiliations:** ^1^ Department of Clinical Nutrition, Faculty of Applied Medical Sciences, King Abdulaziz University, Jeddah, Saudi Arabia.; ^2^ King Fahd Medical Research Center, King Abdulaziz University, Jeddah, Saudi Arabia.

**Keywords:** dropouts, telenutrition, telemonitoring, telehealth coaching, weight-loss

## Abstract

**Background:**

Telehealth programs exhibit strong potential to improve health measures and quality of life among obese and overweight individuals for whom medical nutritional therapy remains a challenge due to poor adherence and dietary compliance. Supporting weight-management programs with dietary interventions or “telenutrition” and integrating telemonitoring and/or telehealth coaching have had a significant positive impact on weight-loss patients achieving their goals in long-term interventions.

**Methods:**

The aim of the current study was to identify the factors leading patients to drop out of a telenutrition weight-loss program, including weekly telemonitoring (total of 36 weeks) and monthly telehealth coaching (total of 6 months). Descriptive qualitative semistructured interviews were held with 10 obese and overweight participants. The data gathered through these interviews were then thematically analyzed through a content analysis.

**Results:**

The findings showed that 50% of participants who dropped out of the study felt pressured at work and/or university. Specifically, 60% reported being influenced by marital responsibilities, and 50% indicated that they did not achieve their goals. Nevertheless, participants who dropped out of the study reported that they were not negatively influenced by family factors and/or financial status. Participants also indicated feeling happy to take part in the program and noted that the diet positively influenced their psychological status. Participants also noted the clarity of instructions and that they were fully motivated during the trial.

**Conclusions:**

The factors associated with dropouts in this study were different from those identified in the literature, given that our weight-loss program was delivered remotely and supported with weekly telemonitoring and monthly telehealth coaching. Despite the dropouts, the interview data highlighted positive factors that could enhance adherence to the dietary program and reduce dropout rates in larger and longer interventions. Future research should highlight the need to develop clear guidelines related to telenutrition programs or other digital health interventions to ensure successful long-term positive outcomes.

## Introduction

Recently, telenutrition weight-loss programs have increased in popularity and offered positive outcomes due to their convenience and easy access among the community.^1–4^ Nevertheless, there is always a 20% possible dropout rate in weight-loss interventions.^5^ Patients dropping out of weight-loss programs remains one of the major factors underlying the failure of obesity therapy, with a prevalence range of 10–80% according to several studies.^6,7^ The term “dropout” refers to a patient’s failure to continue participating in the assigned weight-loss intervention until the end of treatment.^8^ Factors contributing to weight-management dropouts include regaining weight, poor adherence, unsatisfactory results, and increased attrition levels.^6,7^ Evidence also suggests that obesity onset age, gender, body image, full-time employment, unemployment, experience with previous therapy, and a sedentary lifestyle are contributing factors to obesity-treatment dropouts.^6,7^ A prior qualitative study involving 27 participants utilized thematic analysis to identify reasons for dropping out of weight-loss diets. The findings revealed three main themes as follows: personal reasons (such as misunderstandings, lack of motivation, stress, health concerns, mental readiness, and taste preferences), familial and social influences (including social and family problems), and diet characteristics (such as perceived ineffectiveness, high costs, food unavailability, unscientific diets, negative feelings, and unappetizing options). Together, these factors highlight the complex experiences of individuals who have chosen to discontinue their diets.^9^ Another qualitative study has analyzed a larger sample size of 289 participants, 73% of whom were female, with a mean age of 46.68 years and an average dropout rate of 25%. The most significant predictor of dropout was the percentage of weight lost after 1 month; participants who lost 2% or less were 4.99 times more likely to drop out compared with those who lost more than 2%. In addition, among participants at the start of the trial, those aged 50 or younger were 2.07 times more likely to drop out than those older than 50 years old.^10^ A 12-month weight-management program showed that individuals with lower educational levels and higher obesity rates were more likely to drop out of the program.^11^ Recently, it was seen that Cognitive behavioural therapy (CBT) may be an effective approach for treating obesity, often resulting in lower dropout rates compared with other nonbehavioral interventions.^12^ Thus, latest published research have revealed that the main four themes related to dropouts of weight-loss programs are as follows: (1) the challenges faced joining the program and their journey to low-calorie diets, (2) the positive and impactful nature of the program, (3) the interference of life events, and (4) a perceived lack of support from the diet provider.^13^

Generally, the likelihood of losing more weight is significantly associated with greater participation and engagement in virtual and in-person weight-management programs.^14–17^ Understanding the complexity and multiple dimensions of human behavior is also necessary to enhance a patient’s adherence to weight-management programs. Successful lifestyle interventions combine multiple behavioral, environmental, and educational strategies to modify dietary intake and encourage exercise. Indeed, a recent systematic review suggested that combining digital technologies with other established strategies such as follow-ups, health coaching, and feedback might be an effective approach for promoting weight loss.^18^ Moreover, supporting the delivery of dietary modifications with virtual health coaching sessions was associated with improvements to obesity-related outcomes in weight-loss interventions.^19–21^ In health coaching, the patient is the center of the process, whereby coaches educate and help clients promote behavioral accountability, set goals, and plan their next steps to achieve healthy lifestyle changes.^22–24^ As with other weight-loss approaches that coincide with health-related technologies, health coaching can support an individual’s emotions, knowledge, and motivations to change their behaviors and increase their adherence to weight-loss therapy.^25,26^

Previous studies on weight-loss interventions have revealed various factors that may contribute to dropouts such as poor adherence to medication and dietary plans. However, no studies have shown the impact of telemonitoring and telehealth coaching on factors associated with dropouts, for which motivational sessions could overcome related challenges. Moreover, digital health services may encounter dropouts due to technical errors facing clients. Thus, qualitative research investigating the behavioral, psychosocial, and environmental aspects of such dropouts is needed to enhance the success of weight-loss trials.^27^ Under this background, the present study investigates the factors behind dropouts in a telenutrition weight-loss program using weekly telemonitoring and monthly telehealth coaching and determines if such factors are different from those stated in the literature. The goal of this research is to ensure the success of telenutrition weight-loss programs.

## Materials and Methods

### Study design

A previous randomized controlled trial yielded successful outcomes for 18 participants who completed a 6-month telenutrition weight-loss program, through which significant weight loss was achieved.^28^ The intervention group received a hypocaloric diet by a registered dietitian (RD) via telenutrition, in addition to continuous support through weekly telemonitoring and monthly telehealth coaching delivered by an integrative nutrition health coach.^3^ The full study protocol is available in the *British Journal of Nutrition*.^29^ Thus, 10 participants have dropped out from the intervention and were invited to enroll in the present study to investigate the factors underlying their decision to drop out of the program. The study was conducted in the King Fahd Medical Research Center, King Abdulaziz University, in Jeddah. Ethical approval for this research was obtained from the Research Ethics Committee (REC) at the Unit of Biomedical Ethics, Faculty of Medicine at King Abdul-Aziz University, Jeddah, Saudi Arabia (NCBE Registration No: (HA-02-J-008) and (Reference No 527-21), approval date 1 January 2022).

### Semi-structured interview

For this study, we used descriptive semi-structured interviews adapted from a previous study.^9^ These interviews were conducted in a private quiet setting in the King Fahd Medical Research Center. Each participant was assured confidentiality and comfort. Interviews were conducted by two RDs who were working as research assistants in the current study. The length of each interview was approximately 30 min—long enough for more in-depth discourse yet concise enough to maintain participant engagement. A standardized set of 10 open-ended questions was used during the interviews, and participants were first asked to describe their experiences followed by identifying barriers to adherence, which is obtained via specific questions related to the themes identified at the end (Table 1). All interviews were recorded as audio and then transcribed into Microsoft Word. Common themes regarding reasons for dropping out were identified through a thematic content analysis, which elucidated the participants’ experiences and challenges. The content analysis dimensions for dropping out included instructional clarity, motivation to try the diet, home/work pressure or other sources of anxiety, psychological and mental unpreparedness, diet suitability to one’s own tastes, effects of marital status, effects of family problems on experience, goal achievement, financial experiences, the poor availability of specific foods, and the unpalatability of some foods.

**Table 1.** Interview Questions

Question (s) format
1. Was one of the reasons for dropping out from the program the difficulty in understanding the program’s instructions?
2. Was one of the reasons for dropping out from the program the lack of sufficient motivation?
3. Was one of the reasons for dropping out from the program stress and anxiety due to home-related factors or work environment or study struggles in the university?
4. Was one of the reasons for dropping out from the program the mental and psychological unreadiness to follow the program?
5. Was one of the reasons for dropping out from the program your personal dietary preference?
6. Was one of the reasons for dropping out from the program your marital status, for example, being influenced by your partner or by your kids?
7. Was one of the reasons for dropping out from the program related to your social circle, in particular family members?
8. Was one of the reasons for dropping out from the program coming from failure to achieve your goals?
9. Was one of the reasons for dropping out from the program financial reasons?
10. Was one of the reasons for dropping out from the program the unavailability of specific foods?

### Data analysis

Content analysis was used to summarize the semi-structured interviews conducted among the 10 sampled dropouts. The analysis was performed using Microsoft Excel 2019. The content analysis approach conducted^30^ was used previously in a similar qualitative study,^9^ where coding process was iterative and performed line by line through comparative analysis, following six stages of thematic analysis: (1) familiarization with the data, (2) generating initial codes, (3) searching for themes, (4) reviewing themes—where various codes were compared based on their similarities and differences in meaning, (5) defining and naming themes, and (6) producing the final report. Similar codes were grouped into subcategories, and then related subcategories and domains were organized into major categories. There was no significant need for specialized software to study the qualitative data, as there were only 10 participants who provided short, one-sentence responses to each question.

## Results

### Characteristics of the sample

Table 2 presents the sample characteristics. In total, 10 participants were involved in the study, including 5 males and 5 females. The average age of participants was 31.7 years, with most participants (60%) aged between 20 and 29 years. Significantly fewer participants were 30–39 (20%) or 40–49 (20%) years of age. Analyzing the results of the survey, the average of participants’ Body Mass Index (BMI) was 32.89, which is why most of them had obesity. More specifically, 60% of participants were obese, and 40% were overweight.

**Table 2.** Participant Characteristics

Variable	Categories	Frequency	%
Gender	Male	5	50
	Female	5	50
Age	20–29	6	60
	30–39	2	20
	40–49	2	20
BMI	Overweight	4	40
	Obese	6	60

### Summary of the interviews (content analysis)

Table 3 presents a summary of the interview responses. In total, 90% of participants found that the instructions were clear, whereas one participant (10%) struggled to understand them. In terms of motivation, 80% of participants believed that they would adhere to the diet, although one respondent reported feeling less enthusiastic about the diet over time. Ten percent expressed a need for more motivation, and 10% found the diet not motivating at all. When considering external sources of stress, such as home, work, or other anxiety-inducing factors, participants were divided. Half reported experiencing minimal changes, whereas the other 50% felt pressured by factors such as university schedules, transportation issues, and adjusting to a new diet. Interestingly, two participants from the latter group indicated that the diet positively impacted their psychological well-being or made them happier.

**Table 3.** Summary of the Interviews (Content Analysis)

Dimension	Themes	Frequency (*N* = 10)	%	Notes/more info
Instruction clarity	Clear	9	90	
	Unclear	1	10	
Motivation to try the diet	Motivating	8	80	Enthusiasm had reduced after a while
	Require more motivation	1	10	
	Not motivating	1	10	
Home/work/university pressure or other anxiety sources	Pressure felt	5	50	Due to time pressure: e.g., clash with university time, transportation problems, distance from residence, and sudden change of diet
	No change	5	50	Positive feedback: happy, and the diet has changed the psychological condition positively
Psychological and mental unpreparedness	No	10	100	
Diet suitability to own taste	Yes	7	70	
	No	2	20	Dizziness during the trial
	D/K	1	10	Did not start the program due to lack of interest
Effect of marital status	No effect	4	40	
	There was an effect	6	60	Marital responsibilities: changing residence, no time to cook, being influenced by the unhealthy diet of family members or relatives, married and busy
Effect of family problems on experience	No effect	9	90	
	There was an effect	1	10	One said it did affect their psychology a little
Goal achievement	Yes (weight lost)	5	50	Two of them mentioned the exact kg-loss: 2 kg and 4 kg
	No	5	50	Two participants did not start the diet or continue in it; 2 had special medications that caused weight increment or disrupted weight loss; 1 gained weight.
Financial experience	Suitable	7	70	Among them one said that the specialist used to give them many alternatives
	Expensive	1	10	But she used to look for cheaper products
	N/A	2	20	Because they did not actually finish the program
Availability of specific foods being a reason for withdrawal	No	10	100	
Unpalatability of some foods being a reason for withdrawal	No	10	100	

All participants (100%) reported feeling psychologically and mentally prepared for the diet. In total, 70% of participants found the diet agreeable to their tastes, whereas 20% did not. One participant reported dizziness from the diet, and only one participant (10%) did not start the program due to lack of interest. Marital status significantly influenced diet adherence for 60% of participants, who attributed a lack of adherence to changes in residency or the influence of an unhealthy family diet. The remaining 40% reported minimal impacts from marital or familial circumstances. In addition, 90% of participants reported that family-related issues did not affect their diet experience, although one participant did note a modest psychological effect related to family problems. In terms of achieving their goals, 50% of participants reported losing weight, with two individuals specifically noting losses of 2 and 4 kg. However, the other half failed to achieve their weight-loss goals, citing reasons such as not starting the diet, taking medications that hindered weight loss, or gaining weight during the program.

Regarding financial considerations, 70% of participants found the diet affordable, with one patient appreciating the specialist’s willingness to provide alternative options. One participant (10%) found the diet expensive, but managed to find more affordable alternatives. Ultimately, financial concerns were not a significant factor for the 20% of participants who did not complete the program. Finally, all participants (100%) reported that the availability or taste of some foods was not a reason for their withdrawal from the program.

## Detailed Reasons for Dropout

According to the thematic content analysis dimensions, we have organized participants’ reasons according to the dimension investigated in the study (Table 4), which is described below.

**Table 4.** List of Comments Addressed by Participants

Participant #	Comments
1	● Effective social status and excessive preoccupation ● I did not achieve or lose weight because the medicine (Glucophage) reduces the burning rate
2	● There was a little pressure due to moving outside Jeddah ● Some of the products were expensive, but I replaced them with cheaper products
3	● I need more motivation and communication ● I moved to Riyadh and stayed alone for a while, not cooking
4	● There was a little pressure (note: she said this when she was asked about the pressure from work / home) ● The frequent visits did not make me control my eating ● I lost weight in the first few weeks, then I gained weight again during the holidays
5	● It was unclear (note: the participant here means the instructions) unclear instructions ● I did not start the system due to the lack of interest in the program
6	● There was anxiety at the beginning because the food system has changed totally ● Yes, it affected my psychology a little (note: he said this when he was asked about the impact of family problems) ● I did not achieve my goals due to endometriosis because the medications were causing me to gain weight and stop my period
7	● I was the only one with a special diet at home, which affected my discipline
8	● There was time pressure due to clash with university timings ● I did not achieve my goals, and on the contrary, my weight increased
9	● There was transportation pressure ● The effect of work moving to another city
10	● The issue of moving from one house to another and being busy outside home most of the time have affected me

### Instructional clarity

In this subtheme, the unclarity of information was reported and also reported that the research organizers did not provide adequate support, which led to their decision not to start the program.

### Motivation to try the diet

In this subtheme, one participant expressed a desire for increased motivation and better communication from the program, indicating that a lack of encouragement hindered their commitment to the diet.

### Home/work/university pressure or other sources of anxiety

In this subtheme, one participant described the significant pressure he has experienced from both work and home responsibilities.

### Psychological and mental unpreparedness

In this subtheme, one participant was feeling anxious about joining the program due to the complete change in eating habits required, suggesting a lack of mental readiness for the dietary transition.

### Diet suitability to one’s own tastes

In this subtheme, it was indicated that their dietary preferences contributed to nonadherence, highlighting that the diet did not align with their tastes.

### Effects of marital status

In this subtheme, it was noted that living alone during a significant dietary change impacted their discipline, leading to program failure.

### Effects of family problems on experience

In this subtheme, it was reported that one participant is experiencing minor psychological consequences from family problems, which added to their stress and impacted dietary adherence.

### Goal achievement

In this subtheme, although most participants lost some weight during the initial weeks of the program, they subsequently regained it during the holidays, illustrating challenges in achieving long-term weight-loss goals.

### Financial experiences

In this subtheme, one participant found that some dietary products were expensive, but was able to identify cheaper alternatives, indicating that financial considerations influenced their ability to adhere to the diet.

### Poor availability of specific foods and unpalatability of some foods

In this subtheme, transportation issues were identified, which made it difficult to access necessary foods, whereas other participants mentioned that some dietary products were unpalatable, impacting their willingness to follow the diet.

## Discussion

The current study’s main purpose was to identify factors associated with participant dropout in a telenutrition weight-loss program supported by weekly telemonitoring and monthly telehealth coaching. The data underscore several factors leading participants to drop out from the current telehealth intervention, which included participants feeling pressured at work and/or university, marital status, and not reaching their goals. Surprisingly, according to the content analysis of the surveys, a high percentage of participants indicated that they received clear instructions, remained motivated, and were not influenced by family members to drop out. Participants who dropped out of the study also expressed positive takeaways such as feeling happy to participate in the program and positive psychological impacts from the diet. These results are believed to be due to support provided by weekly telemonitoring and monthly telehealth coaching. In contrast, face-to-face dietary interventions without any support from telehealth services yielded opposite results for factors associated with dropouts.

In a similar qualitative study of weight-management trials using 15 categories, most dropouts happened due to personal, familial, or social reasons.^9^ Another study also indicated that dropout rates in weight-management interventions are always associated with family factors,^31^ educational level, and level of obesity.^7^ Furthermore, the literature revealed a significant relationship between dietary attrition and depression, anxiety, and mental health.^32^ In another study, depression, stress, strength, body shape concerns, unemployment, and previous weight-loss attempts were also found to have an impact on dropout rates^33^; these issues could be remedied via telehealth coaching.^34^ Thus, factors contributing to dropping out from telenutrition programs supported with telemonitoring and telehealth coaching differ from those in other types of interventions. This result suggests that telemonitoring and personalized telehealth coaching may provide participants with the motivation and accountability necessary to handle family influence and remain engaged in the program due to the regular feedback provided by health coaches.^35^ When primary care services integrated health coaching to aid their clients, significant weight loss was observed among overweight and obese adults due to increased motivation and ongoing support,^36^ which was also observed in our primary data.^3^

The literature shows that health coaching plays a major role in weight-loss adherence and compliance. Coaching not only has an impact on participant retention in the intervention but also significantly improves medication adherence.^37^ In an experimental study on individuals with chronic obstructive pulmonary disease, health coaching was found to be effective in improving treatment adherence and quality of life.^38^ Consistent with previous studies, digital health coaching was also found to have a crucial impact on adherence, including an increase in remote device adherence, and may help induce a healthy weight-loss rate.^39^ In addition, integrating technology and telemonitoring in weight-management programs can improve motivation, social support, and information sharing to aid in weight loss and lifestyle changes.^40^ Thus, behavioral theory is a vital component when designing any digital health tool utilized by patients to enhance adherence to treatments and support lifestyle changes, thus reducing the rate of dropouts.^41^ Unfortunately, few studies have explored dropouts from digital health interventions. Most of the reasons stated in the literature were related to technological problems, which is believed to be the main barrier for adherence to such interventions. However, we must still identify the main factors contributing to dropouts from digital health interventions. Our study is one of the few using a qualitative interview targeting telenutrition weight-loss programs, which is considered novel and may have strong impact on newly developed telehealth platforms in weight management.

Still, there are several limitations that have occurred in the present study. The main limitation of the present study is its small sample size, as it was a pilot intervention. This restricts the reliability of the findings and recommends future research to cover larger sample size. Another limitation is that despite the novelty of the telenutrition study design, still it restricts applicability of findings on other weight-management interventions that do not use telehealth components. In addition, participants have expressed positive feedback on their experience, despite dropping out, which may conceal the severity of the factors leading to dropout. Furthermore, technological challenges were not reported at all, while it has been confirmed previously in the literature as barriers to adherence in digital health interventions, but the present study did not consider specific technological challenges faced by participants, which could have provided additional insights into dropout reasons.

## Conclusions

This study revealed the factors contributing to dropout in a telenutrition weight-loss program, which included marital status, workplace pressure, and not achieving one’s goals. Notably, these factors differed from those commonly associated with usual dietary interventions. The results of the telenutrition program demonstrated that support through telemonitoring and monthly telehealth coaching had positive impacts on the influence of family, the clarity of instructions, and motivation. As telenutrition and telehealth interventions continue to grow, it is essential to develop effective integrative strategies to improve dietary compliance, program adherence, and dropout rates in weight-management programs. The results obtained from the present study suggest several future perspectives to reduce the number of dropouts in weight-loss programs as follows: (1) utilizing technology in weight-loss programs, (2) improving the integration of telemonitoring using telehealth devices, (3) improving the integration of telehealth coaching by adding more sessions to enhance dietary compliance and adherence levels, (4) identifying participants that are at higher risk of dropping out, and (5) designing comparative randomized controlled trials to investigate different telehealth strategies and identify the best approaches to enhance dietary compliance and reduce dropout rates.

## Data Availability

The data presented in this study are available on reasonable request from the corresponding author: ooaeid2@kau.edu.sa

## Abbreviations Used

BMI: body mass index
CBT: cognitive behavioural therapy
REC: research ethics committee
