# Supplementary material for: Identifying Factors Contributing to Dropouts in a Pilot Telenutrition Weight-Loss Program: A Qualitative Study
Source: Telemed Rep. 2024 Dec 11;5(1):393–401. doi: 10.1089/tmr.2024.0071 (PMC11671310; doi:10.1089/tmr.2024.0071)
Supplement: Supplementary Data S1 [file tmr.2024.0071_supp_datas1.pdf]

We certify that the following article

Identifying Factors Contributing to Dropouts in a Telenutrition Weight Loss Program: A qualitative study

Noura Eid

has undergone English language editing by MDPI. The text has been checked for correct use of grammar and common technical terms, and edited to a level suitable for reporting research in a scholarly journal.
MDPI uses experienced, native English speaking editors. Full details of the editing service can be found at
► https://www.mdpi.com/authors/english.

MDPI
Multidisciplinary
Digital Publishing
Institute

Basel, Switzerland
October 2024

AUTHORservices

english-86640
